# Classification of Fricative Consonants for Speech Enhancement in Hearing Devices

**DOI:** 10.1371/journal.pone.0095001

**Published:** 2014-04-18

**Authors:** Ying-Yee Kong, Ala Mullangi, Kostas Kokkinakis

**Affiliations:** 1 Department of Speech Language Pathology & Audiology, Northeastern University, Boston, Massachusetts, United States of America; 2 Bioengineering Program, Northeastern University, Boston, Massachusetts, United States of America; 3 Department of Speech-Language-Hearing, University of Kansas, Lawrence, Kansas, United States of America; University of California, Irvine, United States of America

## Abstract

**Objective:**

To investigate a set of acoustic features and classification methods for the classification of three groups of fricative consonants differing in place of articulation.

**Method:**

A support vector machine (SVM) algorithm was used to classify the fricatives extracted from the TIMIT database in quiet and also in speech babble noise at various signal-to-noise ratios (SNRs). Spectral features including four spectral moments, peak, slope, Mel-frequency cepstral coefficients (MFCC), Gammatone filters outputs, and magnitudes of fast Fourier Transform (FFT) spectrum were used for the classification. The analysis frame was restricted to only 8 msec. In addition, commonly-used linear and nonlinear principal component analysis dimensionality reduction techniques that project a high-dimensional feature vector onto a lower dimensional space were examined.

**Results:**

With 13 MFCC coefficients, 14 or 24 Gammatone filter outputs, classification performance was greater than or equal to 85% in quiet and at +10 dB SNR. Using 14 Gammatone filter outputs above 1 kHz, classification accuracy remained high (greater than 80%) for a wide range of SNRs from +20 to +5 dB SNR.

**Conclusions:**

High levels of classification accuracy for fricative consonants in quiet and in noise could be achieved using only spectral features extracted from a short time window. Results of this work have a direct impact on the development of speech enhancement algorithms for hearing devices.

## Introduction

A common configuration of hearing loss is high-frequency hearing loss, which affects the perception of speech sounds that have mostly high-frequency (2000–10000 Hz) energy, such as fricative consonants. There are a total of nine fricative consonants in English: /f, θ, s, ∫, v, ð, z, З, h/, and eight of them (all except for/h/) are produced by partially obstructing the airflow through the oral cavity. These fricative consonants differ in terms of the point of constriction in the vocal tract (i.e., place of articulation) – labiodental/f, v/; interdental/θ, ð/; alveolar/s, z/; and palatal/∫, З/. Within each place, the fricatives differ in regard to the absence (voiceless) or presence (voiced) of the vocal fold vibration – voiceless/f, θ, s, ∫/; and voiced/v, ð, z, З/. Note also that the greatest spectral difference between voiced and voiceless phonemes is mainly at the low-frequency region below 1000 Hz where the voiced fricatives have higher energy at low frequencies compared to their voiceless counterparts. Given the large number of fricative consonants in the English language, perceptual deficits in this class of sounds could severely reduce oral communication especially in noisy listening environments.

### Effect of Hearing Loss on Fricative Perception

Previous studies have reported that there are multiple cues to the perception of fricatives, including the spectral differences in the fricative noise and lower frequency energy in the transition from fricative noise to the adjacent vowel [1]–[3]. Zeng and Turner [2] reported that adults with hearing loss rely primarily on fricative noise for the discrimination of voiceless fricatives, and their conclusion was further supported by recent work by Stelmachowicz et al. [4]. It has been shown that reduced audibility and spectral resolution at high frequency due to hearing loss significantly impaired listeners' ability to discriminate fricative consonants [5].

### Signal Processing to Enhance Perception of High-Frequency Speech Components

Signal processing techniques that increase the audibility of fricative sounds or spectral differences among fricatives could enhance speech perception performance in hearing-impaired listeners. To enhance listeners' ability to perceive high-frequency fricative content, recent research reported a significant benefit for speech recognition in hearing-impaired individuals with high-frequency amplification above 4 kHz compared to amplification only up to 4 kHz. For example, Stelmachowicz et al. [4] provided spectral cues up to 9 kHz to hearing-impaired adults and children with moderate to moderately severe hearing loss (i.e., between 40 and 70 dB HL) at 2 and 4 kHz. They reported that although the perception of the fricative/s/in quiet improved with increasing stimulus bandwidth, fricative perception performance remained significantly poorer for hearing-impaired than for normal-hearing listeners.

While extended high-frequency amplification could provide benefit for aidable hearing loss, for individuals who have severe-to-profound high-frequency hearing loss, signal processing strategies that lower high-frequency speech components to a lower-frequency region, such as frequency compression [6] or frequency transposition [7] have been recommended. While most algorithms utilized in hearing aids lower the high-frequency phonemes by a fixed ratio or by a constant for all speech sounds, others operate conditionally on nonsonorant consonants (i.e., fricatives, affricates, and stops) for frequency lowering [8]–[9].

Recent work in our laboratory [10]–[11] explored a method of frequency lowering that targets nonsonorant consonants and also enhances the spectral contrasts of the frequency-lowered fricatives. This speech enhancement method involves classification of fricative consonants followed by spectral shaping of the frequency-lowered signals based on the classification results. Here, we describe the conceptual framework of this algorithm, which prompted the investigation of fricative classification in the current study, along with a summary of the perceptual results from hearing-impaired listeners. The aforementioned frequency-lowering algorithm is described in more detail in Kong and Mullangi [11].

*A Vocoder-Based Frequency-Lowering System with Spectral Enhancement*: The vocoder-based frequency-lowering algorithm developed by Kong and Mullangi [10]–[11] is divided into analysis and processing stages. During the analysis stage, input signals are first bandpass-filtered into a number of frequency bands. The filtered signals are then subjected to an analysis that separates speech sounds into two classes, sonorants and nonsonorants. Only the nonsonorant sounds, which contain aperiodic high-frequency energy, will proceed to a second analysis that separates high-frequency frication sounds into three groups. Once classified, the nonsonorant sounds undergo frequency lowering via channel vocoding by which the amplitude of the high-frequency bands is used to amplitude modulate bands of low-frequency noise, which is added to the original speech signal. To enhance the spectral differences of the transposed signal for fricative perception, the spectrum of the low-frequency noise used in the system is determined by the classification results. With an earlier prototype of the system with fricative classification accuracy at about 88% on VCV syllables in quiet, Kong and Mullangi [11] reported that listeners with steeply-sloping high-frequency hearing loss received significant benefit for place-of-articulation perception for fricative consonants in quiet.

Since the spectral enhancement method described above selectively processes specific classes of phonemes, its success hinges on the accuracy of classification of speech sounds. The goal of this study is to further investigate acoustic features and classification methods that could separate fricative consonants into separate classes that differ in place of articulation with high levels of accuracy in quiet and also in noise. Although dynamic or relational features including relative amplitude between the fricative consonant and the adjacent vowel(s), duration of the fricative consonants, and relative amplitude of fricatives, have been shown to contribute to the correct classification of place of articulation in fricatives, only static acoustic features extracted within a very short time window were considered in the current study to allow for a real-time signal processing implementation in hearing devices. Previous research has shown that processing delay of less than 10 msec is necessary for hearing aids in order to preserve the sound quality and to prevent the user's perception of his or her own voice [12]–[13]. Thus, in this study, we used a short time window of 8 msec, which is well within the limit set by the industry. Among the static acoustic properties, combinations of spectral features including spectral slope, spectral peak location, and four spectral moments [14]–[17], were found to be robust features for separating fricatives into three groups. Previous studies and our own preliminary analyses did not find static features that could reliably separate the non-sibilant fricatives further into two groups (labiodental and inter-dental fricatives). It was shown that normal-hearing listeners use formant transition cues to perceptually discriminate labiodental and interdental fricatives. Also, at high frequencies, both voiced and voiceless fricatives have the similar patterns of spectral shape that are distinctive among fricatives differing in place of articulation. In addition, this work considers more realistic communication situations often encountered by users of listening devices, which include (1) speech recognition in quiet and in noisy situations at different signal-to-noise ratios (SNRs) and (2) variations of acoustic characteristics due to talker differences.

In this paper, we present and compare classification results of fricative sounds using a support vector machine (SVM) algorithm. Various acoustic static features, including the previously reported spectral features (i.e., spectral moments, peak, and slope), bandpass-filtered outputs, magnitude of fast Fourier Transform (FFT), and Mel-frequency cepstral coefficients (MFCCs) are investigated. Our results show high levels of fricative classification performance with spectral features extracted from a relatively short time frame. This suggests that the frication noise contains sufficient information about the identity of the fricative consonants, and that our classification methods have potential clinical applications on the future development of speech enhancement in hearing devices.

## Methods

### Speech materials

The classification experiments were performed on fricative consonants extracted from a TIMIT database [18], which contains a total of 6,300 continuous sentences spoken by 639 speakers from eight dialect regions in the United States. The sentences were recorded in a noise-free environment with a sampling rate of 16 kHz and 16-bit resolution. The TIMIT database is divided into training and testing sets. The core testing set in the database has a total of 168 speakers, resulting in a total of 1,344 sentences. In our experiments, the audio files were first scaled to have equal root-mean-squared (RMS) amplitude across all sentences in both the training and testing set. Additionally, the RMS-equalized TIMIT sentences were corrupted with two different types of noise. The level of the noise was adjusted depending on the pre-determined SNR test conditions for each experiment. For the training set, clean speech was corrupted with speech-shaped noise (SSN) at +10 dB SNR. To approximate the long-term average spectra of adult speech [19], the SSN was created by lowpass-filtering white noise with a first-order Butterworth filter using a cutoff frequency of 800 Hz. The use of this generic type of SSN allows for greater generalization of the classifier to other real-life listening situations. For the testing set, clean speech was corrupted with a 12-talker speech babble noise [20] at seven different SNRs (+20, +15, +10, +5, 0, −5, −10 dB). The rationale behind using different types of noise was to create a mismatch in the training and testing environments. The choice of multi-talker speech babble noise was motivated mainly by its ecological relevance. For each sentence and for each SNR condition, the clean speech was mixed with a different time segment of the speech babble noise. Each fricative was then extracted from the clean and noisy sentences using the TIMIT phone labels and transcription boundaries.

### Feature extraction for fricatives differing in place of articulation

Several types of static acoustic features have been shown to be different among the three classes of fricatives. Motivated by existing literature [14], [15], and [17], we examined six spectral features, including four spectral moments (i.e., mean [M1], variance [M2], skewness [M3], kurtosis [M4]), dominant spectral peak location (P), and spectral slope (S) for a subset of fricative tokens in the TIMIT training set, which were used for classification as will be described in the “Classification Procedure” section. Acoustical and statistical analyses were performed to confirm that these features are indeed discriminative among the fricative groups.

Spectral analyses on the six acoustic features were performed on clean speech using a 128-point FFT in the frequency range from 1kHz to 8 kHz. The upper frequency of 8 kHz was limited by the sampling rate of the recorded speech materials. Information was extracted from a randomly chosen 8-msec segment using a hamming window of the fricative tokens, and acoustical analyses were performed on each of these 8-msec segments. The mathematical description of the four moments can be found in Forrest et al. [21] and Maniwa & Jongman [17]. Spectral peak location (P) is defined as the frequency that corresponds to the highest amplitude peak of the FFT spectrum. Spectral slope is computed here as the difference between the highest and lowest amplitude of the FFT spectrum divided by the corresponding difference in the frequency. Each acoustic measurement was first examined to determine its contribution in the fricative classification using a Kruskal-Wallis test. This non-parametric test was used because the assumption of normality was not met for some of the acoustic features examined. Table 1 shows the measured median values for each feature for each fricative type along with statistical results. As indicated by the *p*-values in this table, the values of the acoustic features were significantly different between the three groups of fricatives. Hence, combinations of these features were included in the classification experiments.

**Table 1:** Median values and Kruskal-Wallis test results (*p*-values) for six acoustic features for the training set.

Measurements	/f, θ, v, ð/	/s, z/	/∫, З/	*p*-value
M1 (Hz)	3,989	4,909	4,117	< 0.0001
M2 (MHz)	3.89	2.47	2.55	< 0.0001
M3	0.31	−0.11	0.58	<0.0001
M4	−0.95	−0.14	−0.39	<0.0001
P (Hz)	1,750	4,625	3,250	<0.0001
S	−0.008	0.011	−0.006	<0.0001

Besides the well-documented acoustic features described above, we investigated additional three groups of features described below. Again, for training of the classifiers, the acoustic features were extracted from a randomly chosen 8-msec segment of the fricative tokens using a hamming window, and acoustical analyses were performed on each of the 8-msec segments. The upper frequency was restricted to 8 kHz limited by the sampling rate of the recorded speech.

#### (1) Two versions of MFCCs – MFCC(3) and MFCC(13)

MFCC(3) denotes the first three coefficients of the 3 cepstra without derivatives and MFCC(13) denotes 13 cepstra without derivatives. MFCC(3) represents the first three coefficients of MFCC(13). The MFCCs were extracted using the VOICEBOX toolkit [22].

#### (2) Gammatone filter outputs

Two feature vectors of outputs of bands of Gammatone filters. One vector consisted of outputs of 14 filters [Gammatone(14)] at the high-frequency above 1 kHz, and the other consisted of 24 filter outputs [Gammatone(24)] for the wide frequency spectrum above 0.1 kHz. Gammatone(14) is a subset of Gammatone(24).

#### (3) Magnitude spectrum of FFT

The magnitudes of 128-point FFT at each frequency bin were calculated in the frequency range from 0.1 to 8 kHz.

Given that the numbers of dimensions are high for the Gammatone(24) and FFT feature vectors, dimensionality reduction techniques that project the high-dimensional feature vector onto a lower dimensional space were used to enable the classifier to achieve improved generalization through: (1) eliminating redundant dimensions that may not convey reliable information for the classification, (2) determining a manifold that exhibit maximal information about the class label, and (3) avoiding over-fitting from the classifier [23]. For signal processing in real-time, decreasing the number of dimensions of the feature vector could result in a considerable reduction of the overall processing time. For a high-dimensional feature vector 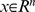, the aim is to determine a smooth mapping function ***f***:R^n^→R^m^ where m<n such that the reduced dimensional feature vector is *y =* ***f(x)***. In this study, we investigated both linear and nonlinear projections to achieve the optimal manifold.

For linear dimensionality reduction (LDR), a commonly-used variable reduction technique referred to as principal component analysis (PCA) was used to map the data to a lower-dimensional space [24]. The data is transformed to orthogonal axes corresponding to the direction of the maximum variance in the original data space such that the first few dimensions of the new space account for the majority of variance in the data. For a random vector 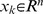, the mean of each of the features is adjusted to 0 using Eq. (1):
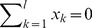
(1)


where *l* is the number of points in the dataset.

The covariance matrix of *x* given by Eq. (2) is computed to obtain the eigenvalues and eigenvectors:
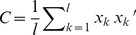
(2)


To reduce the number of dimensions from ***n*** to ***m***, the eigenvectors corresponding to ***m*** largest eigenvalues of the covariance matrix are used for approximating the original ***n***-dimensional vector. ***m*** is determined by the number of eigenvalues that exceed a pre-determined threshold.

For nonlinear dimensionality reduction (NLDR), we adapted a projection technique using a kernel PCA [25]. A kernel PCA first projects the data into a feature space by calculating the kernel matrix (described below) such that a nonlinear function is applied to the original data with a reduced computational cost. A PCA can be then performed on the projected data. Let Ф be a nonlinear function that projects the random vector ***x_k_*** into a feature space *F* to obtain Ф(***x_k_***). A kernel matrix (*K*) is calculated as in Eq. (3). 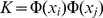
(3)


where *i* and *j* index the row and the column in the matrix, respectively, and that both *i* and *j* vary from 1 to *l*.

To obtain a centered kernel matrix with a zero mean, a Gram matrix is computed using Eq. (4).
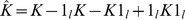
(4)


where 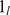
is an *l*x*l* matrix with all elements equal to 1/*l*.

Once projected to the nonlinear space, procedures for dimensionality reduction follow the principles of PCA as described above. The eigenvectors computed from the Gram matrix are taken as the kernel principal components.

### Classification Procedure

A one-stage classification procedure with a SVM algorithm was used for classifying fricatives into three groups. The MATLAB library LIBSVM described in Chang & Lin [26] was employed to perform a multi-class classification using a one-against-one strategy.

We used a subset of fricatives contained in the TIMIT training set to train the classifiers, and all fricatives in the TIMIT core testing set during the testing of the classifiers. The fricatives chosen for the training phase were sampled from a subset of speakers in all dialects in the TIMIT training set. The numbers of training tokens were similar across fricative groups. For training, acoustic information from a randomly chosen 8-msec segment of the fricative tokens was used. Given that the spectrum of the frication noise is relatively stable over the entire fricative consonant [14], random samples from each fricative token should provide a good representation of the fricatives for a large number of speakers while keeping the size of the training set manageable. The resulting numbers of fricative samples were 1,864 for the labio/inter-dental fricative group, 1,648 for the alveolar fricative group, and 1,455 for the palatal fricative group. Feature vectors were extracted from each of the 8-msec speech segments. During the training phase, the clean speech tokens and the tokens that were corrupted with SSN at +10 dB SNR in the training set were passed on as inputs to the SVM classifier.

In our preliminary study with a smaller number of speech tokens, we investigated a number of kernel types and kernel functions. We concluded that the C-support vector classification (C-SVC) kernel type and the radial basis kernel function (RBF) yielded the best performance. Thus, we used the C-SVC and RBF in this study. During a grid search in the training phase, the C parameter of the C-SVC and the gamma parameter of the RBF were selected using a cross validation procedure [27].

After the SVM classifier was trained, classification was performed on all the fricative tokens in the TIMIT testing set. Similar to the training phase, classification was made for every 8-msec segment of the fricative consonants. Classification was performed on speech tokens in quiet, as well as on speech tokens corrupted with speech babble noise at different SNRs. To summarize the performance of the classifiers, classification accuracy reported below was determined on a consonant-by-consonant basis, in which the majority vote was taken as label for the sequence [28]–[30]. This means that the fricative group corresponding to the most frequently selected group across the 8-msec frames concluded the classification of the consonant. Overall classification accuracy for the fricative tokens in the testing set was calculated as the proportion of the fricatives correctly identified. This approach, as opposed to the calculation of the accuracy frame-by-frame, allows for a comparison of our results with findings from other studies [15], [28].

## Classification Results

### Comparisons of Features

**Figure 1:**
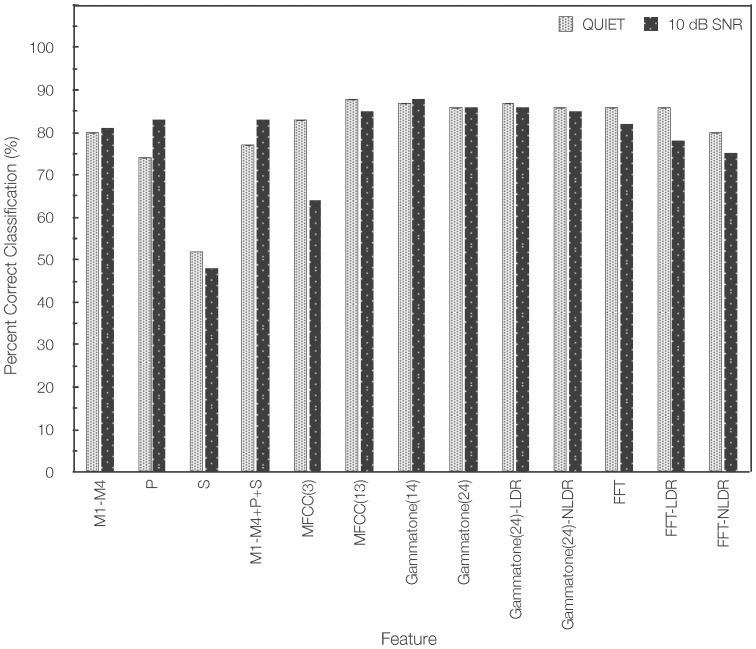
Overall classification accuracy in quiet and at +10 dB SNR with different of features.

Figure 1 shows the overall classification accuracy achieved using various features. As supported by the statistical results, the knowledge-based acoustic features (i.e., moments, peak location, and slope) yielded an above chance-level performance for both quiet and +10 dB SNR conditions. On the basis of Bernoulli fluctuations at the performance levels of 50%–90% and over 6,500 trials, the width of 95% confidence interval for each measure is less than 2.5 percentage points. The results showed that including the peak location and slope features did not significantly enhance the classification accuracy of moment features, as the M1-4+P+S condition (77% in Q and 83% in noise) produced similar percent correct scores to the M1-4 condition (80% in Q and 81% in noise).

There was no considerable difference in classification performance among the MFCC(13), Gammatone(14), Gammatone(24) features, with percent accuracy at 86%–88% in quiet and 85%–88% in noise. In comparison, classification accuracy using the FFT feature was slightly lower in noise (82%). The results of MFCC(3) were significantly lower in both quiet (83%) and noise (64%) conditions than those obtained by MFCC(13). For Gammatone(24), classification results were similar with and without dimensionality reduction, and the two reduction methods (linear vs. nonlinear) produced similar classification accuracy. It is noted that the resulting dimensions were 6 and 7 for the linear and nonlinear reduction, respectively. In contrast, dimensionality reduction decreased the classification accuracy for the FFT feature with the nonlinear reduction (reduced to 5 dimensions) showed a greater detrimental effect than linear reduction (reduced to 4 dimensions).

Overall classification accuracy was generally higher with MFCC, Gammatone, and FFT features when compared to that obtained with the spectral moments, peak, and slope features. For example, classification accuracy was higher with Gammatone(14) compared to the other six acoustic features combined (i.e., M1-4+P+S) by 10 percentage points in quiet and 5 percentage points in noise.

### Classification at Different Signal-to-Noise Ratios

**Figure 2:**
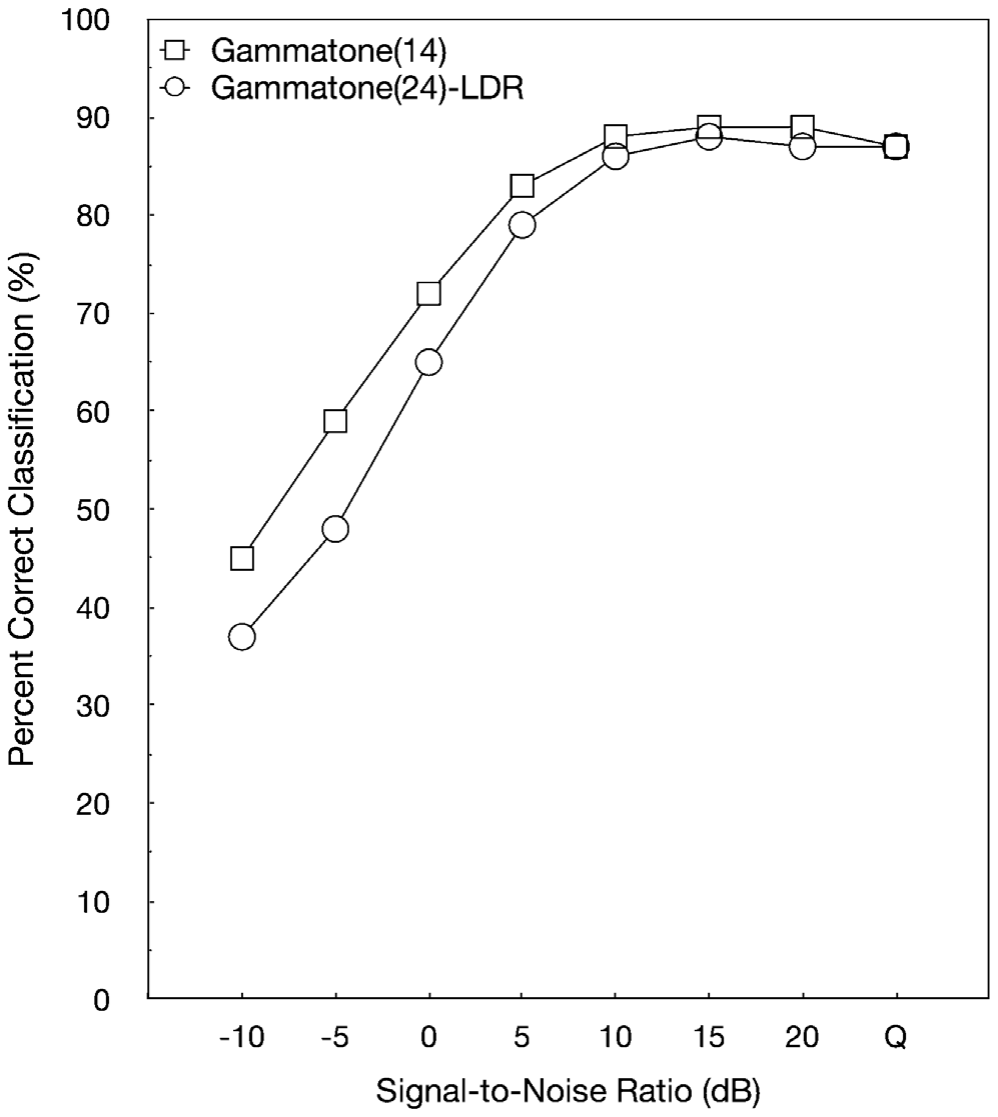
Overall fricative classification accuracy in quiet and at seven SNRs ranging from +20 to −10 dB using Gammatone(14) and Gammatone(24)-LDR.

Given that classification accuracy at +10 dB SNR was high for Gammatone features and Gammatone filters are commonly used in auditory modeling, a follow up classification experiment was performed using Gammatone(14) and Gammatone(24)-LDR on fricative consonants that were corrupted with speech babble noise at SNRs ranging from +20 down to -10 dB. Similar to the experiment described above, the SVM classifier was trained with both clean speech and speech materials that were corrupted with SSN at +10 dB SNR. Figure 2 plots the overall fricative classification accuracy. Results showed that classification performance was on average 9 percentage points higher for Gammatone(14) than for Gammatone(24)-LDR at lower SNRs from 0 dB to -10 dB, suggesting that the discriminative features for classification of three groups of fricatives are primarily centered at high frequencies above 1 kHz. Using the Gammatone(14) feature, classification accuracy remained greater than 80% for SNR conditions from +20 dB SNR down to +5 dB SNR, despite the fact that the noise for training (SSN) and testing (12-talker babble) were different. As the level of the noise increased, more sibilant fricatives/s, z, ∫, З/were identified as non-sibilant fricatives/f, v, θ, ð/. Table 2 shows the confusion matrices for each group of fricatives in quiet and for three noise levels (+15 dB SNR, +10 dB SNR, and +5 dB SNR) using the Gammatone(14) feature.

**Table 2:** Confusion matrices for fricative classification in quiet and at three SNRs using Gammatone(14).

Clean speech: 87% accuracy	Detected as/f, θ, v, ð/	Detected as/s, z/	Detected as/∫, З/
/f, θ, v, ð/	85%	11%	4%
/s, z/	1%	87%	12%
/∫, З/	1%	10%	89%
*+15 dB SNR: 89% accuracy*	Detected as/f, θ, v, ð/	Detected as/s, z/	Detected as/∫, З/
/f, θ, v, ð/	95%	4%	1%
/s, z/	3%	89%	8%
/∫, З/	3%	13%	84%
*+10 dB SNR: 88% accuracy*	Detected as/f, θ, v, ð/	Detected as/s, z/	Detected as/∫, З/
/f, θ, v, ð/	97%	2%	1%
/s, z/	6%	87%	7%
/∫, З/	5%	15%	80%
*+5 dB SNR: 83% accuracy*	Detected as/f, θ, v, ð/	Detected as/s, z/	Detected as/∫, З/
/f, θ, v, ð/	98%	2%	0%
/s, z/	14%	81%	5%
/∫, З/	13%	17%	70%

### Summary and Discussion

The present study examined acoustic features for classification of fricative consonants that differ in place of articulation. Among the features examined, MFCC(13), Gammatone(14), and Gammatone(24) produced similar classification results in quiet and at +10 dB SNR. A subsequent analysis showed that Gammatone(14) yielded a high level of classification accuracy even at challenging noise conditions, down to +5 dB SNR.

### Classification Accuracy

For clean (uncorrupted) speech, the MFCC(13), Gammatone, and FFT features achieved 86%–88% correct classification, a similar level of performance to that reported in the literature [14]–[16]. For example, using a knowledge-based decision-tree-like algorithm, Ali et al. [15] reported 91% accuracy for classifying fricative consonants into three groups. Previous studies reported results similar to the present study using an artificial neural network [15] and a linear discriminant analysis [14], [16]. However, relational features, such as relative amplitude between the fricative consonant and the neighboring vowel were used in these studies. Without the relational feature, Ali et al. [15] reported an 87% correct classification for fricatives in quiet when the classifier was trained and tested on the same set of speech materials. Recently, Frid & Lavner [28] resorted to an SVM algorithm and a set of 15 features that included spectral peak, spectral moments, and MFCC(3) to classify fricatives into four groups and reported an accuracy of 85%. It is noted that only four voiceless fricatives/f, θ, s, ∫/were considered in the Frid & Lavner [28] study, without including the voiced fricatives/v, ð, z, З/.

For noisy speech, the SVM algorithm with the Gammatone(14) feature achieved correct classification greater than 80% for SNRs from +20 dB to +5 dB SNR. It is important to note that (1) both clean and noisy speech were used for the training, (2) the noise was different between training and testing, and (3) the classifier was trained with noisy speech at only one SNR condition (+10 dB SNR), but was tested on seven SNR conditions (from +20 to −10 dB SNR). In other words, the training and testing environments were mismatched.

### Effect of Dimensionality Reduction

Two methods of dimensionality reduction – linear and nonlinear projections – were investigated to lower the dimensions of the high-dimensional feature vectors for our dataset. The purpose of this was to determine the most discriminative dimensions and also reduce the processing time for classification. Our results showed that, depending on the feature vector, classification performance with nonlinear projection was either similar to that with linear projection [Gammatone(24)] or slightly lower than the classification accuracy obtained with linear projection (FFT).

When comparing performance with the feature vector containing only high frequencies, our results showed that classification performance was on average 9 percentage points higher for Gammatone(14) than for Gammatone(24)-LDR at lower SNRs from −10 dB to 0 dB, suggesting that the discriminative features for classification of three groups of fricatives in speech babble noise are primarily at high frequencies above 1 kHz.

### Importance of Spectral Information above 8 kHz

The TIMIT sentences used in the current study have a relatively low sampling rate of 16 kHz, which could have a negative effect on the fricative classification accuracy. Previous research reported spectral differences among three groups of fricatives for frequencies above 8 kHz [10], [16], and [31]. Thus, we hypothesize that classification accuracy would improve for speech materials with a higher sampling rate that allows for data analysis at high frequencies, because a possible cue for discrimination between alveolar and palatal fricatives is the high-frequency fall-off, which is usually above 8 kHz. Another cue for discrimination between sibilant and non-sibilant fricatives is the spectral slope above 8 kHz [10].

To demonstrate the importance of high-frequency information above 8 kHz on fricative classification, we conducted a small-scale classification study using speech materials with a sampling rate of 44.1 kHz. The recorded speech materials included 8 fricative stimuli in/vowel-consonant-vowel/utterances with three vowels (/a, i, u/), resulting in a total of 24 syllables. These stimuli were spoken three times (three repetitions) by each of 14 speakers (six male adults, six female adults, one male child age 11, and one female child age 11), resulting in a total of 1,008 tokens. The adult speakers were taken from the recordings in Shannon et al. [32] and the two child speakers were recorded in our laboratory. The RMS amplitude of all stimuli was equalized to the same value. We divided the stimuli into two sets: training set and test set. The training set contained speech stimuli from four adult males and four adult females. The test set contained the stimuli from the remaining speakers (two male adults, two female adults, one male child, and one female child). Similar to the experiments described above, the classifier was trained with clean and noisy speech with SSN at +10 dB SNR, and was tested with clean and noisy speech with multi-talker babble noise for a wide range of SNRs. Acoustic features used for the classifier included outputs of Gammatone filters above 1 kHz. In one condition, the recorded speech materials were down-sampled to 16 kHz, which limits the upper frequency for analysis to 8 kHz. In another condition, the originally recorded stimuli at a sampling rate of 44.1 kHz were used, which allows for acoustic analysis for frequencies above 8 kHz.

**Figure 3:**
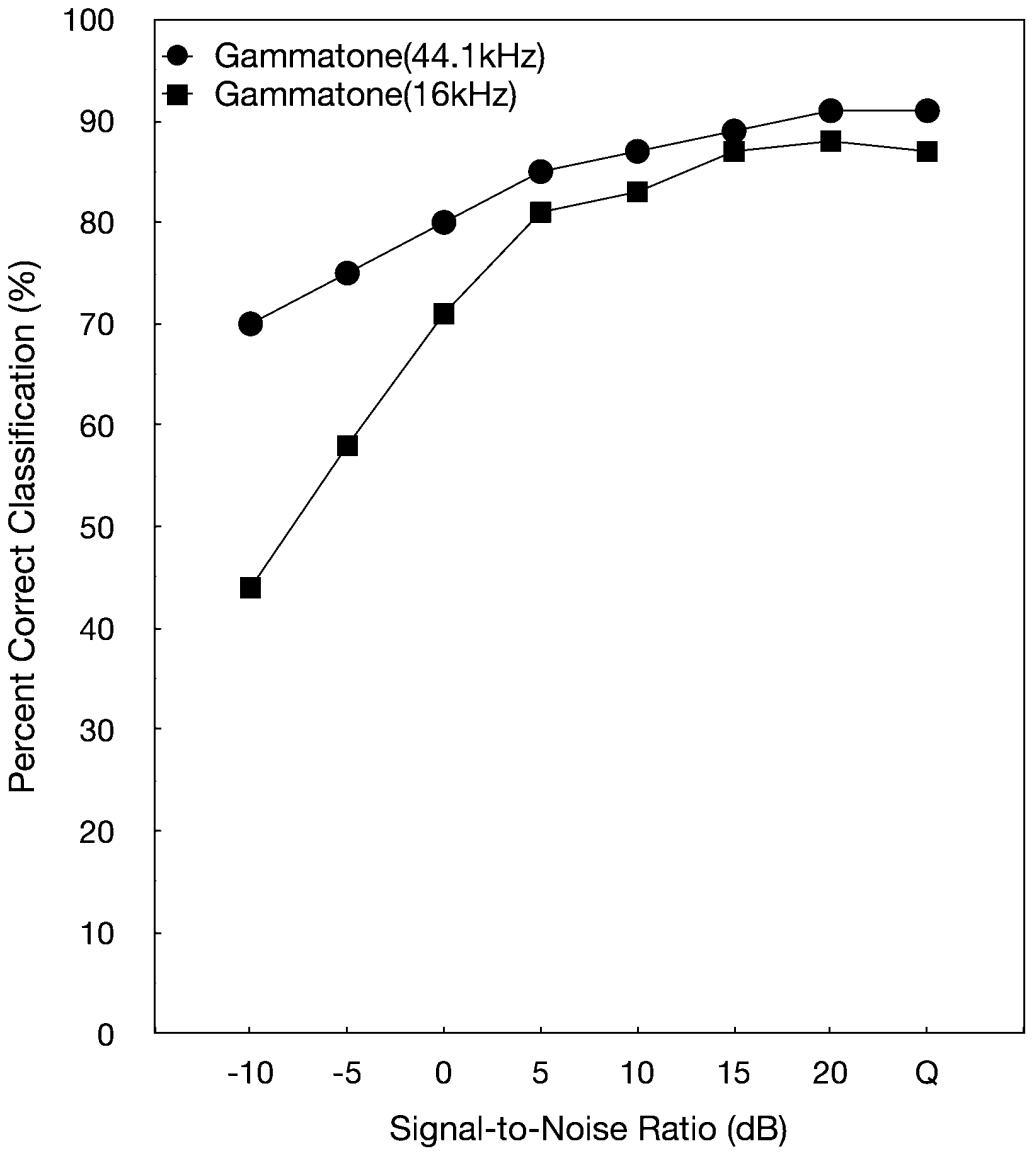
Overall fricative classification accuracy observed in quiet and at seven SNRs ranging from +20 to −10 dB of speech materials with sampling rates equal to 16 kHz and 44.1 kHz.

Figure 3 shows the percent classification accuracy in quiet and for SNRs from −10 dB to +20 dB for the two sampling rate conditions. Classification accuracy increased as the sampling rate increased from 16 to 44.1 kHz, and improvement was greater with an average increase of 17 percentage points for lower SNRs at 0, −5, and −10 dB.

### Application to Signal Processing for Hearing Devices

The results in the present study suggest that the proposed classification method that uses only static acoustic features extracted from an 8-msec time frame could potentially be implemented in real-time for potential use in hearing aid devices and other auditory prostheses. The earlier prototype of our frequency lowering algorithm [10], [11] used different methods (i.e., pre-determined thresholds, linear discriminant analysis) and different acoustic features to classify fricative consonants, and the classification accuracy was lower than that reported in the current study. In preliminary perceptual studies conducted with hearing-impaired listeners, discrimination of fricative consonants after frequency lowering improved with the spectral enhancement method [11]. In listening conditions with a low SNR, the perception of fricative consonants could be further improved by resorting to the features and classification method described here.
